# The complete chloroplast genome and phylogenetic analysis of *Salix triandra* from China

**DOI:** 10.1080/23802359.2019.1674743

**Published:** 2019-10-15

**Authors:** Dongyang Wu, Yupeng Wang, Li Zhang, Lijun Dou, Linming Gao

**Affiliations:** a College of Information Science and Technology, Nanjing Forestry University, Nanjing, Jiangsu, China;; b Information Center of Nanjing Forestry University, Nanjing, Jiangsu, China;; c Computer Center of Information Science and Technology, Nanjing Forestry University, Nanjing, Jiangsu, China

**Keywords:** *Salix triandra*, chloroplast genome, phylogeny

## Abstract

*Salix triandra* is a great willow for bees and an excellent choice for living willow structures. In this study, we assembled and annotated the complete chloroplast (cp) genome sequence of *S. triandra*. The whole cp genome is 155,821 base-pairs (bp) in size, which comprises one small single copy (SSC) region of 16,223 bp and one large single copy (LSC) region of 84,532 bp separated by a pair of inverted repeats (IRs) of 27,533 bp. There are 131 genes, including 86 protein-coding genes, 37 tRNA genes, and 8 rRNA genes. Phylogenetic analysis with the Neighbour-joining method indicates that *S. triandra* is closely related to *S. tetrasperma*. The complete cp genome will facilitate the biological studies in the order Malpighiales in future.

*Salix triandra* (common names: almond willow, almond-leaved willow) is a species of willow native to Western and Central Asia and Europe. *S. triandra* has immense economic value because it provides bees with abundant pollen and nectar early in the year (Farkas and Zajácz 2007). *Salix triandra* belongs to flowering plants of Salicaceae family, which contains 56 genera and about 1220 species summarized by the Angiosperm Phylogeny Group (APG) (Christenhusz and Byng 2016). In this paper, the complete cp genome sequence of *S. triandra* is characterized for further phylogenetic studies of the family Salicaceae.

The total genomic DNA was extracted from fresh leaves of *Salix triandra* collected in Maoer Mountain (China; N45°19′40.60″, E127°37′9.03″) using TakaRa MiniBEST Plant Genomic DNA Extraction Kit (Tokyo, Japan). Voucher specimen was deposited in the Key Laboratory of Forest Genetics and Biotechnology, Ministry of Education, Nanjing Forestry University (DB447). Then, the whole-genome sequences were conducted on PacBio RS II Sequencing Instrument (Pacific Bioscience, USA) by Nextomics Biosciences (Wuhan, China). After controlling quality and correcting of 17.6 G raw data, the remaining high-quality reads were assembled with Falcon version 3.0 (Chin et al. 2016). There are many similar cp genomes of homologous species available, which usually as reference genomes to acquire the order of contigs (Wang et al. 2018). So we used BLASTN (Camacho et al. 2009) to isolate cp contigs of *S. triandra* based on *S. suchowensis* (NC_026462.1). The annotation of *S. triandra* cp genome was conducted with DOGMA (Wyman et al. 2004) with manual check and the physical map was drawn by OGDRAW (Lohse et al. 2007).

The complete cp genome of *S. triandra* (GenBank accession number: MK722343) is 155,821 bp, and shares the common feature of comprising an LSC region of 84,532 bp, an SSC region of 16,223 bp, and two IRs region of 27,533 bp. The total length of protein-coding genes is 91,877 bp, accounting for 58.96% of the complete cp genome and encoding 26,646 amino acids. The overall GC content is 36.65%, higher than those of LSC (34.39%) and SSC (30.94%) regions, but lower than those of IRs regions 41.79%). The cp genome possesses 131 functional genes, including 86 protein-coding genes, 8 rRNA genes, and 37 tRNA genes. Among these genes, three genes (rps12, clpP, ycf3) have two introns, and other 17 genes have one intron each. There are 18 genes (7 protein-coding genes, 7 tRNA and 4 rRNA genes) duplicated in the IR regions.

To identify the phylogenetic position of *S. triandra*, 24 cp genomes (excluding *S. triandra*) of the family Salicaceae were downloaded from National Centre for Biotechnology Information (NCBI). These 25 cp genomes sequences were aligned with ClustalW (Thompson et al. 2002). The phylogenetic tree (Figure 1) based on 70 protein-coding genes using the neighbour-joining method was reconstructed by MEGA7 (Kumar et al. 2016). The phylogenetic analysis showed that *S. triandra* is evolutionarily closest to *S. tetrasperma* with 100% bootstrap value.

**Figure 1.**
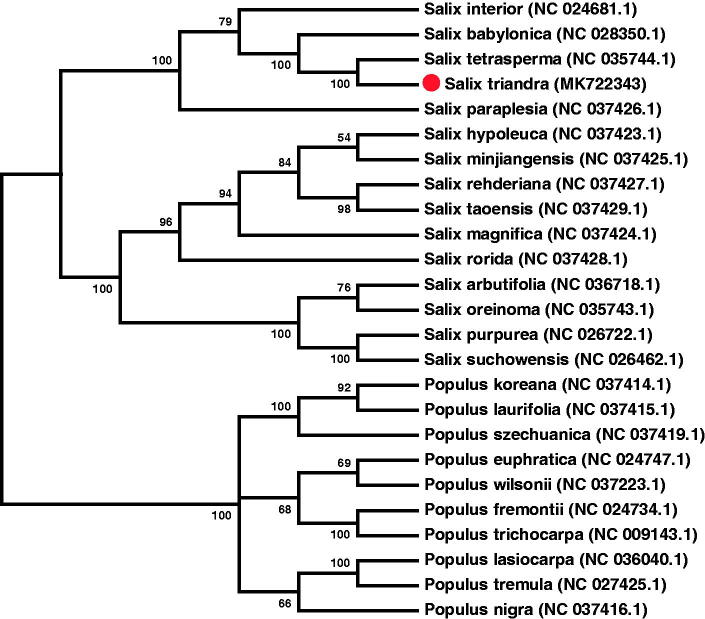
Phylogenetic tree with neighbour-joining method was constructed by MEGA7. The 70 protein-coding genes of 25 cp genomes from the Salicaceae family were aligned by ClustalW. The bootstrap values (indicating on the branches) were based on 1000 replicates and the accession number is list after each species name.
